# A light field‐based method to adjust rounded leaf end MLC position for split shape dose calculation correction in a radiation therapy treatment planning system

**DOI:** 10.1120/jacmp.v13i6.3937

**Published:** 2012-11-08

**Authors:** Jia‐Ming Wu, Tsair‐Fwu Lee, Chung‐Ming Kuo, Ching‐Jiang Chen, Kuan‐Yin Hsiao, Shyh‐An Yeh

**Affiliations:** ^1^ Department of Information Engineering I‐Shou University Taiwan; ^2^ Department of Radiation Oncology E‐Da Hospital Kaohsiung Taiwan; ^3^ Institute of Radiological Science Central Taiwan University of Science and Technology Taichung Taiwan; ^4^ Medical Physics & Informatics Lab Department of Electronics Engineering National Kaohsiung University of Applied Sciences Kaohsiung Taiwan; ^5^ Department of Medical Imaging and Radiological Sciences I‐Shou University Taiwan

**Keywords:** multi‐leaf collimator, rounded leaf end, split shape, dose correction

## Abstract

We present an analytical and experimental study of split shape dose calculation correction by adjusting the position of the round leaf end position in an intensity‐modulated radiation therapy treatment planning system. The precise light field edge position ($X_{\text{tang}.p}$) was derived from 50% of the central axis dose created by nominal light field using geometry and mathematical methods. Leaf position ($X_{\text{mlc}.p}$), defined in the treatment planning system for monitor unit calculation, could be derived from $X_{\text{tang}.p}$. Offset (correction) could be obtained by the position corresponding to 50% of the central axis dose minus the $X_{\text{mlc}.p}$ position. For SSD from 90 cm to 120 cm at 6 MV and 10 MV, the 50% dose position was located outside of $X_{\text{mlc},p}$ in the MLC leaf position range of +8 cm to ‐8 cm, where the offset correction positively increased, whereas the offset correction negatively increased when the MLC leaf position was in the range of ‐12 cm to ‐8 cm and +20 cm to +8 cm when the 50% position was located inside $X_{\text{mlc},p}$. The monitor unit calculation could provide underdosage or overdosage of 7.5% per mm without offset correction. Calibration could be performed at a certain SSD to fit all SSD offset corrections. With careful measurement and an accurate offset correction, it is possible to achieve the dose calculation with 0.5% error for the adjusted MLC leaf edge location in the treatment planning system.

PACS number: 87.53.Tf, 87.55.x, 87.55.D, 87.55.dk

## I. INTRODUCTION

For IMRT treatment technique, MLC systems are available on most commercial linear accelerators, and many of these MLC systems utilize designs with rounded leaf ends to improve the dose profile on the geometry penumbra and the transmission penumbra. The general designs of rounded leaf‐end MLC system have been described by many researchers.^1^, ^8^ These MLC design considerations result in differences between the MLC 50% isodose points and the projected light field edge locations. These differences need to be corrected before patients treatment monitors are calculated by treatment planning system. Radiation field size is defined as the lateral distance between the 50% isodose lines at a reference depth. This definition is practically achieved by a procedure called the beam alignment in which the field‐defining light is made to coincide with the 50% isodose lines of the radiation beam projected on a plane perpendicular to the beam axis and at the standard source‐to‐surface distance (SSD 100 cm) or source‐to‐axis distance (SAD 100 cm). The geometry of projecting split light field and its radiation field of the moving rounded leaf‐end MLC need to be measured and implemented into computerized treatment planning system. Because the coincidence between the dose 50% position and the spilt field cannot be taken for granted with the nondivergent geometry that is found in the curved leaf linear type of collimator system,^(9)^ the dose 50% position must be verified during MLC system acceptance. When patients' treatment monitor units are calculated in split MLC situation, the dose 50% position correction in treatment planning system should be calibrated precisely to avoid underdosage or overdosage of patients' treatment. Radiation dose profile measurement of leaf position has been early made in the commissioning of MLC system in accelerator, usually at 100 cm SSD. In this work, we will illustrate some of the specific issues that should be considered if we want to make precise dose calculation of split shape associated with rounded leaf‐end MLC system at different treatment SSD.^(10)^

## II. MATERIALS AND METHODS

**Figure 1:**
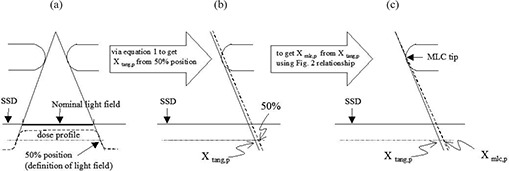
The summary of measurement procedures in this study: (a) the projection of the nominal light field at an SSD of 100 cm was adopted for dose profile measurements; (b) the precise light field edge ($X_{\text{tang}.p}$) was derived from 50% of the central axis dose by geometry and mathematical methods (Eq. (1)); (c) $X_{\text{mlc}.p}$ (leaf position was defined in the treatment planning system) can be derived from $X_{\text{tang}.p}$; then correction “Offset” could be obtained by subtraction of the 50% of the central axis dose position from the $X_{\text{mlc}.p}$ position.

The work presented here was performed on an Elekta precise linear accelerator (Elekta, Stockholm, Sweden) with a dual photon energy of 6 MV and 10 MV. The Pinnacle v8.6 treatment planning system (Philips Healthcare, Andover, MA) was used for photon dose calculations after the linear accelerator clinical data had been implemented. Dose profiles of multi‐leaf collimated fields were measured and the calculation results of treatment planning system were compared. The procedures of this study are summarized in Fig. 1.

**Figure 2:**
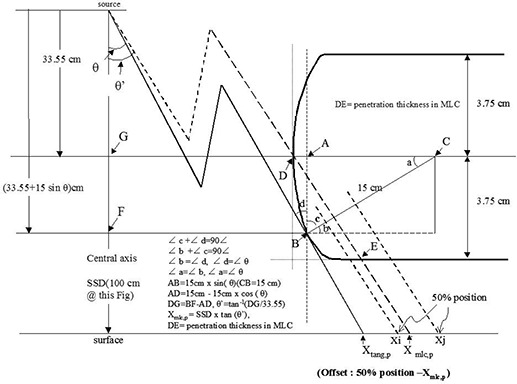
Schematic drawing of a rounded leaf MLC placed in a linear accelerator. Leaf edge position of the tangential field $\left(X_{\text{tang},p}\right)$, planning leaf position $\left(X_{\text{mlc},p}\right)$, and offset (50% dose position minus position of $X_{\text{mlc},p}$) are indicated. The track of radiation in the MLC to the projected 50% dose position is located at $X_{j}$ (outside of $X_{\text{mlc}.p}$) or $X_{i}$ (inside $X_{\text{mlc},p}$) depending on the leaf position from the central axis.

All penumbra profiles were measured at a certain visual light field (nominal light field) and at an SSD of 100 cm to obtain the position receiving 50% of the central axis dose. The projection of the nominal light field at an SSD of 100 cm was adopted for dose profile measurements, but the dose profile from the nominal light field edge could not quantitatively determine the geometry of the tangential edge ($X_{\text{tang},p}$) for the derivation of $X_{\text{mlc},p}$ (planning leaf position); therefore, the precise light field edge ($X_{\text{tang}.p}$) was derived from 50% of the central axis dose by geometry and mathematical methods (Eq. (1)). Leaf position was defined in the treatment planning system for monitor unit calculation ${(}_{\text{Xmlc}.p}$, the intersection of the source to the leaf tip with a certain SSD surface in Fig. 2). This point could then be derived from $X_{\text{tang}.p}$. Once the $X_{\text{mlc}.p}$ was decided,

the correction “Offset” could be obtained by subtraction of the 50% of the central axis dose position from the $X_{\text{mlc}.p}$ position.

### A. Geometry specifications

#### A.1 Nominal light field

The approximate size of the actual light field for patient treatment and dose profile measurements.

#### A.2 Xtang.p – light field tangential edge position in decimal value

According to Fig. 2, the linear accelerator MLC rounded leaf end determines $X_{\text{tan}.p}$, which is the intersection of the source to point B with the isocenter plane at different SSD. The $X_{\text{tan}.p}$ is used quantitatively to describe the leaf edge, while the nominal light field (visualized light field) edge is used qualitatively by humans to check the boundary of the treatment area.

#### A.3 Xmlc.p – treatment planning leaf position

The position of the tip (D in Fig. 2) of the leaf projected to the extended SSD plane. Patient dose calculations are based on this point in the treatment planning system.

#### A.4 The 50% dose position

The attenuation of radiation in MLC should be enough to obtain 50% of the central axis dose. When MLC moves near to or away from the central axis, the 50% dose position might be located at $X_{j}$ (outside of $X_{\text{mlc}.p}$) or $X_{i}$ (inside $X_{\text{mlc}.p}$), respectively. It depends on the radiation attenuation in MLC (denoted as $\bar{DE}$ in Fig. 2); usually, $X_{j}$ is outside of $X_{\text{mlc}.p}$ when the field size is less than 16 cm (‐8 cm to +8 cm). The 50% dose position will be located at $X_{i}$ (inside $X_{\text{mlc}.p}$) when the leaf position is from ‐12 cm to +20 cm, excluding from ‐8 cm to +8 cm.

#### A.5 Direction of the MLC

When the MLC travels away from central axis (the field size becomes large), the direction is denoted as positive (“+” in all figures). When the MLC travels closer to, or crosses over the central axis, the direction is denoted as negative (“‐” in all figures).

#### A.6 Relationship of geometry and radiation position

The linear accelerators used in this study were equipped with MLCs for IMRT dose delivering devices. Many investigators have described the design and characteristics of MLC.^(11)^ According to a study by Topolnjak and Van der Heide,^(11)^ one analytical approach for optimizing the leaf design of a multi‐leaf collimator assesses the relationship between the light field size edge position ($X_{\text{tang}.p}$, lp in Eq. (1)) and the 50% dose position ($X_{i}$ or $X_{j}$ in Fig 2, $P_{t50}(1p)$ in Eq. (1)). The radiation penumbra model (denoted as Eq. (1) in this study) used in this study was modified by this analytical approach equation for deriving the leaf edge position of the tangential split shape($X_{\text{tang}.p}$) from 50% dose position($P_{t50,\text{SSD}}(\text{lp})$) at a certain SSD; the parameters in Eq. (1) are source to MLC mid‐plane distance (c), source to surface distance (F), attenuation coefficient (μ), radius of leaf tip (R), and leaf position (lp). Leaf position (lp) is also denoted as $X_{\text{tang}.p}$ in Fig. 2.

### B. Measurement devices

Radiation field size data were measured using water phantom scans and GAFCHROMIC films (International Specialty Products, Wayne, NJ). Computer‐controlled water phantom scanning systems (PTW MP3 Water Phantom Systems, PTW‐Freiburg, Germany) utilizing $0.015\;{\text{cm}}^{3}$ ion chambers (Type 31016 ion chambers, PTW‐Freiburg, Germany, measurement point 1.3 mm behind the chamber tip, active cylinder length 3.6 mm, diameter 2.9 mm) were used for measurements with each field size. Measurements were made with the ion chamber at the isocentric plane with water depths of 10 cm. All profiles were normalized on the central axis, except in cases where the leaves were tangentially near to — or crossed — the central axis, and which were normalized at the center of the irradiated area. The field sizes were defined at the 50% intensity points relative to the central value of the profile. With the same field sizes, random measurement of water phantom scans and GAFCHROMIC film method techniques were performed for the comparison of multi‐leaf collimated field size profiles. These field sizes measured with film agreed with the same field sizes measured with water tank scans to within 0.2 mm. After verifying that the film method achieved the same results as the water tank method, film proved to be more efficient, so we chose to use the GAFCHROMIC film method for this study on 50% of the central axis dose measurements.

### C. Film measurement

#### C. 1 Film setup

The field sizes adopted in this experiment were generated by the nominal field size and positioned at leaf positions from +20 cm with 1 cm increments to ‐12 cm (crossover central axis ‐12 cm). GAFCHROMIC film was exposed to individual rectangular fields defined by the MLC. Each film was placed at extended SSD from 90 cm with increments of 10 cm to 120 cm at a depth of 10 cm phantom. The field size was defined at the 50% intensity points relative to the central value of the profile.

#### C.2 Film measurement and process

The films were exposed perpendicularly to the radiation beam in a solid water phantom. The 50% dose positions were measured by GAFCHROMIC EBT 2 film (ISP Technology, Inc., Wayne, NJ, Log F04090901, expiry date: April, 2011). A double exposure technique(^12–17^) was adopted for these measurements. This was performed by giving each film an initial dose of 2 Gy and measuring the optical density before experimental irradiation was given. A variation of 2% was observed in the optical density for the films used in the experiment due to nonuniformity in the dose response.^18^, ^22^

A calibration to convert from raw scanner signal to dose was done. This was achieved by placing 5 cm solid water phantom slabs on top of the 3.0 cm×3.0 cm film pieces with a field size of 10 cm×10 cm, a source‐to‐axis distance (SAD) of 100 cm, and by irradiating them with step size of 10 cGy in the dose range from 10 cGy to 150 cGy under an Elekta Precise medical linear accelerator machine. In this study, we used an Epson Expression 10000XL flat‐bed document scanner (US Epson, Long Beach, CA). Film pieces are scanned using the VariSoft software (PTW, Freiburg, Germany), with the maximum OD range and all filters and image enhancement options turned off. Once the scanner is turned on, it is important to perform a preview operation in transmission mode and then allow the scanner to warm up for half an hour. This operation turns on the upper lamp used for transmission mode and allows its temperature to stabilize. The films were scanned in the 48‐bit RGB mode, 16 bits per color, and saved as tagged image file format TIFF image files.

The first step in the protocol is to scan the unexposed pieces of film five times. Multiple scans have been performed in order to remove the scanner noise by subsequent averaging of the scanned images. Once the five images of the unexposed film pieces have been acquired, blank scans are taken, again five times, over the same scanning region as the previously acquired images with the film pieces. This allows one to correct for “defective” pixels, defined as pixels that differ in intensity from the blank unattenuated signal, which is equal to 2.

In general, scanned images with irradiated films will have a scanning region different from that of the unirradiated film pieces. Therefore, for the removal of the defective pixels in irradiated film images, five blank scans are made again of the irradiated film scanning region. Then the processing of the images was the identification of defective pixels. Since two glass plates are in the optical pathway, in addition to the examined films, the system can exhibit many imperfections. This identification was performed over the resulting images obtained by averaging five successive scans of the empty bed, for both unirradiated and irradiated film images. We found that the percentage of faulty pixels was smaller than 0.4%.

The net optical density (OD) of a point on the film is given by $OD=\log_{10}(S_{0}/S)$ where $S_{o}$ is the background (i.e., the scanner signal for an unexposed film) and *S* is the scanner signal for the film at the point of interest.

### D. Derivation of offset correction

#### D.1 To derive the 50% dose position into Xtang,p

The parameters relating to the position $P_{t50}(1p)$ is given by the radiation penumbra model (see Appendix A) in Eq. (1):

(1) $$P_{t50}(lp)=F\cdot \tan\left\{\begin{array}{l} \left(\arctan\left(\frac{c\cdot \frac{lp}{F}+R\cdot \cos\left(\arctan\left(\frac{lp}{F}\right)\right)+\frac{lp}{F}\cdot R\cdot \sin\left(\arctan\left(\frac{lp}{F}\right)\right)}{c}\right)\right)-\\ \left(\arcsin\left(\frac{\sqrt{R^{2}-{\left(\frac{\ln(0.5)}{2.\mu }\right)}^{2}}}{\sqrt{c^{2}+{\left(c\cdot \frac{lp}{F}+R\cdot \cos\left(\arctan\left(\frac{lp}{F}\right)\right)+\frac{lp}{F}\cdot R\cdot \sin\left(\arctan\left(\frac{lp}{F}\right)\right)\right)}^{2}}}\right)\right) \end{array}\right\}$$

In Eq. (1), lp means *FS*/2 and is $X_{\text{tang}.p}$ in Fig. 2. The leaf position (lp) could be calculated with the measured 50% dose position at a certain SSD using this equation. The $X_{\text{mlc}.p}$ could then be derived from $X_{\text{tang}.p}$ (leaf position (lp)) for offset correction.

#### D.2 Attenuation coefficient: μ

The coefficients of attenuation $\mu =0.772\;{\text{cm}}^{‐1}$ and $\mu =0.843\;{\text{cm}}^{‐1}$ were used for correspondence photon energies of 10 MV and 6 MV, respectively (Bureau of Radiological Health 1970).

#### D.3 To derive Xtang,p into Xmlc,p

Once a light field size was defined, $X_{\text{tang},p}$ was the light field edge in precise decimal value. Once $X_{\text{tang}.p}$ was defined, Θ' could be derived from Θ to get $X_{\text{mlc},p}$.

#### D.4 The geometric derivation of Xmlc,p

According to Fig. 2, ∠Θ was determined from $X_{\text{tang}.p}$ as follows:

$$\begin{array}{rl} & ∠c+∠d=90^{\circ }\\ & ∠b+∠c=90^{\circ }\\ & ∠b=∠d\\ & ∠d=∠\Theta \\ & ∠a=∠b\\ & ∠a=∠\Theta \\ & \bar{AB}=15cm\times \sin\theta \\ & \left(\bar{CB}=15cm\right)\\ & \bar{AD}=15cm-15cm\times \cos\theta \\ & \bar{DG}=\bar{BF}-\bar{AD}\\ & \theta^{\prime }=\tan^{-1}(\frac{\bar{DG}}{33.5})\\ & X_{mlc,p}=SSD(cm)x\tan(\theta^{\prime })\\ & \bar{DE}=penetration\;thickness\;in\;MLC \end{array}$$

#### D.5 Offset definition

Patient treatment monitor unit calculation was based on $X_{\text{mlc},p}$ in the treatment planning system. The $X_{\text{mlc},p}$ should be assigned to measure the 50% dose position for precise treatment monitor unit calculation in split shape situation. The definition of adjustment offset is as follows: Offset=the 50%; dose position ‐ the position of $X_{\text{mlc},p}$.

## III. RESULTS

### A. Film results

**Figure 3:**
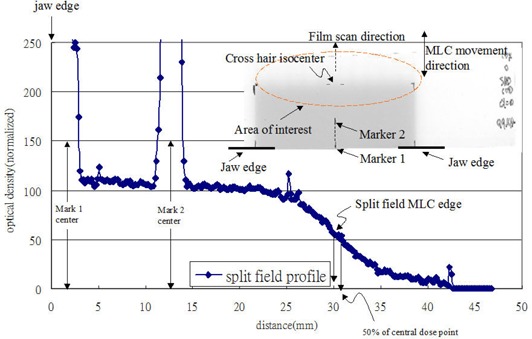
Graphics of the film setup and drawing of the split field profile for 50% of the central axis dose position measurement at an SSD of 100 cm with a 10 MV photon beam. Mark 1 and mark 2 were used to identify the position receiving 50% of the central axis dose.

In Fig. 3, one of film results of the split field is shown. Marker 1 on the film was 30 mm away from the crosshair isocenter and the MLC edge traveled to abut the crosshair on irradiation. After converting the optical density to a dose distribution on the film, the position receiving 50% of the central axis dose was 31.38 mm away from marker 1 instead of 30 mm away. The 1.38 mm discrepancy was due to the photon transmission and scatter effect.

### B. Fifty percent dose position of split shape with an extended SSD

**Figure 4:**
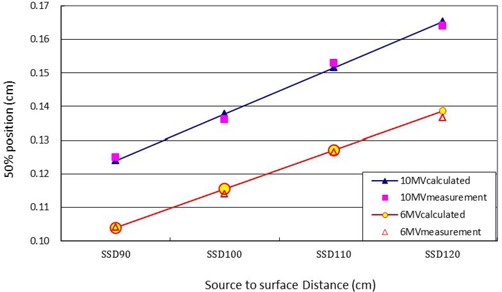
Graphics of the split field 50% dose position at different SSD of the central axis at 10 MV and 6 MV. For a split field MLC leaf edge projecting to an enlarged SSD plane, the number of scatter photons increased under the MLC blocked area, which led the 50% of the central axis dose position to move toward the MLC shadow. With greater photon energy and a larger SSD, the 50% dose position moved closer to the MLC shadow.

Figure 4 shows the result of 50% dose position of the split field at extended SSD at 10 MV and 6 MV. The results indicate that a longer SSD and higher photon energy increases the distance of the 50% dose position from the crosshair isocenter.

### C. Offset correction at an SSD of 100 cm

**Figure 5:**
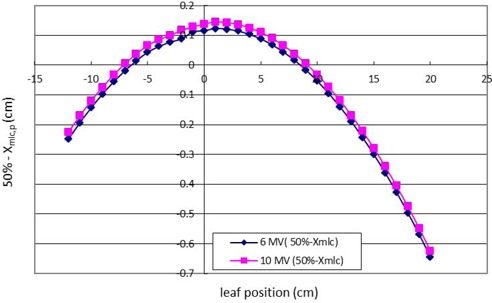
Graphics of offset correction of different photon energies at an SSD of 100 cm. The offset correction is from positive to negative due to the reversed position between the 50% dose point and $X_{\text{mlc},p}$.

After the 50% dose position was measured from the size of the visual light field (nominal light field), the associated quantity of $X_{\text{tang}.p}$ (lp) could be obtained by using Eq. (1). The $X_{\text{mlc}.p}$ could be derived from Fig. 2 by implementing $X_{\text{tang}.p}$, and the final offset corrections were calculated. Figure 5 shows the result of the 50% dose position minus $X_{\text{mlc}.p}$ vs. leaf position at 6 MV and 10 MV at SSD 100 cm.

### D. Offset correction with an extended SSD at 6 MV

**Figure 6:**
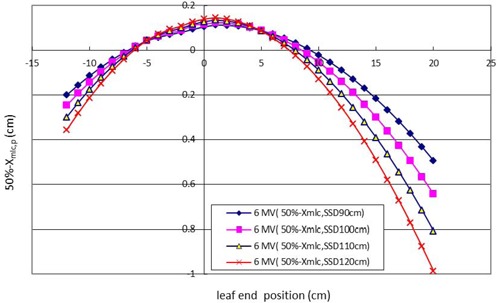
Graphics of offset correction of an extended SSD at 6 M V. The offset correction is affected not only by scatter photon contributions to the central axis in split field edge, but also at the distal positions of +20 cm and ‐12 cm at SSD longer than 120 cm. The longer SSD necessitates more offset correction.

Figure 6 shows the offset corrections which could be obtained by changing the F (SSD) in Eq. (1). In the figure, when the leaf traveled close to the central axis in the range from ‐8 cm to +8 cm, the offset corrections were positive. When the leaf traveled away from the central axis in the range from ‐8 cm to ‐12 cm and +8 cm to +20 cm, the offset corrections became negative and greater in proportion.

### E. Offset correction with an extended SSD at 10MV

**Figure 7:**
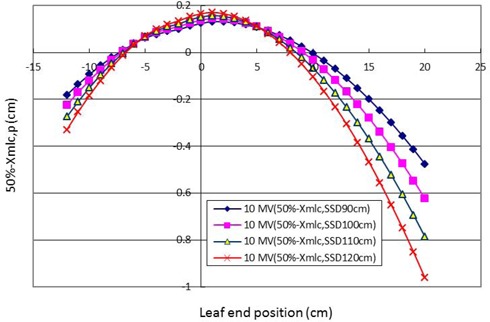
Graphics of offset correction of an extended SSD at 10 MV. The offset correction is affected by a similar pattern as that at 6 MV.

Figure 7 shows the offset correction with an extended SSD at 10 MV. The offset corrections shown in this figure are similar to those found at 6 MV.

## IV. DISCUSSION

The precise leaf edge position of the tangential split field $\left(X_{\text{tang},p}\right)$ could be derived by the measured 50% dose position from the mathematical model and can be used to obtain the defined planning leaf position $\left(X_{\text{mlc},p}\right)$. The offset (the 50% dose position minus the planning leaf position) could be determined for the purpose of accurate monitor unit calculation, as shown in Fig. 2. The relationship between $X_{\text{tang},p}$ and $X_{\text{mlc},p}$ was further analyzed with a multileaf collimator geometry model at different treatment SSD (F in Eq. (1)). Figure 2 shows $X_{\text{tang},p}$, $X_{\text{mlc},p}$, and the 50% central axis dose position ($X_{i}$ or $X_{j}$). If the MLC round leaf travels close to the central axis, the 50% dose position gains attenuation and will project outside of $X_{\text{mlc},p}$ on $X_{j}$. As the MLC round leaf travels away from the central axis, the 50% dose position will project inside $X_{\text{mlc},p}$ and gain less attenuation, as shown on $X_{i}$. This offset adjustment can be of importance in clinical situations of split fields to determine overdosage or underdosage at extended treatment SSD.

The $X_{\text{mlc}.p}$ was calculated by a mathematic analytical model using Eq. (1), using the 50% dose position measured by GAFCHROMIC films. Figure 3 shows one of the films in the experimental setup, and the profile result of the split light field edge and the position receiving 50% of the central axis dose at an SSD of 100 cm with a 10 MV photon beam. Mark 1 was delineated by the jaw edge 30 mm from the crosshair isocenter, and mark 2 (this mark was used for a double check of position setting accuracy) was 15 mm away from the center of mark 1. Fifty percent of the central axis dose can be found via the profile through an optical density to dose conversion; this moves away from the central axis toward the MLC shadow due to side scatter of photons and electron contributions.

This result of film measurement showed the positions of the 80% dose and the 20% dose at 26 mm and 34 mm, respectively. The width of the split field penumbra of the 80% dose to the 20% dose was approximately 8 mm, and changed with the rate of dose gradient by 7.5% per mm at an SSD of 100 cm with a 10 MV photon beam. Monitor unit calculation in the treatment planning system is completely decided upon by the selected point on the split field penumbra curve. The selected point on the descending or ascending portion from the 50% dose to the 20% dose, or from the 80% dose to the 50% dose, will lead to overcalculated or undercalculated results for monitor units.

For a split field of MLC leaf edge projected to longer SSD, there was more photon scatter under the MLC blocked area, which led to the 50% dose position of the open half beam moving toward the MLC shadow. With greater photon energy and a longer SSD, a greater shift of 50% dose position of the open half beam into the MLC shadow was observed (Fig. 4).

The 50% dose position was larger at 10 MV than at 6 MV because sidescatter electrons have more penetration at 10 M V. When patients treatment monitor units are calculated in a split field situation, the offset (50% dose position minus $X_{\text{mlc},p}$) correction should be calibrated precisely to avoid underdosage or overdosage of patients. The calculated monitor units for treatment will be less than the desired dose and lead to underdosage due to overcorrection because the 50% of the central axis dose point (for monitor unit calculation purposes) passes through the ascending portion from 50% to 80%. The 50% point (for monitor unit calculation purposes) passes through the descending portion from 50% to 20%, so the underestimated output selected in this region will lead to overcalculated monitor units and result in overdosage. The offset correction should be adjusted by the extended SSD accordingly, because the 50% dose position was increased from an SSD of 90 cm to 120 cm both at 6 MV and 10 MV in this study. Figure 5 shows the offset (50% dose position minus $X_{\text{mlc},p}$) correction at 10 MV and 6 MV with an SSD of 100 cm. The 50% dose position was located outside of $X_{\text{mlc},p}$, since more attenuation leads to the positive offset correction in the range from +8 cm to ‐8 cm, whereas the negative offset correction is in the range from ‐12 cm to ‐8 cm and from +20 cm to +8 cm because the 50% dose position is located inside $X_{\text{mlc},p}$.

This study shows not only that scatter photons contribute to the central axis in the split shape edge to move the 50% dose position away from the central axis towards the MLC shadow, but also demonstrates the same photon scatter phenomenon at distal MLC positions of +20 cm and ‐12 cm at the extended SSD of 120 cm. We observed that with longer SSD, more offset corrections are shown (as seen in Fig. 6). In comparison to Fig. 6, the phenomenon of positive and negative offset correction gradually increased in a similar pattern with an extended SSD at 10 MV, as shown in Fig. 7.

Table 1 shows how to convert the 50% of the central dose position to its relative light field tangential edge position in decimal value (lp in Eq. (1) and is $X_{\text{tang},p}$ at Fig. 2, not nominal light field) in order to obtain the treatment planning leaf position $\left(X_{\text{mlc},p}\right)$ for offset correction.

**Table 1:** This table demonstrates the calculation results of the offset correction of 10 MV and 6 MV photon beams at an SSD of 100 cm. The offset corrections for extended SSD can be calculated by a procedure similar to that used in this table.

*1*	*2*	*3*	*4*	*5*	*6*	*7*	*8*	*9*	*10*	*11*	*12*	*13*
*Nominal Field Size(N cm)*	*10 MV Rad 50% Position (cm)*	$10MVX_{\mathrm{tang},p(cm)}$	*Derived θ*	Source to F[33.55+15×sin(θ)](cm)	FB [(source to F)×tan (θ)](cm)	AD[15 cm‐15×cos(θ)](cm)	*GD (FB ‐ AD) (cm)*	*[arctan (GD/33.55cm)]*	$X_{mlc,p}from\theta '(cm)$	$10\;MV(50\%‐X_{mlc,p})(cm)$	*6MV Rad 50% Position (cm)*	$6\;MV(50\%‐X_{mlc,p})(cm)$
‐12	‐11.820	‐11.816	‐6.84	31.76	‐3.81	0.11	‐3.92	‐0.12	‐11.60	‐0.22	‐11.844	‐0.25
‐11	‐10.836	‐10.826	‐6.28	31.91	‐3.51	0.09	‐3.60	‐0.11	‐10.67	‐0.17	‐10.860	‐0.19
‐10	‐9.849	‐9.827	‐5.71	32.06	‐3.21	0.07	‐3.28	‐0.10	‐9.73	‐0.12	‐9.872	‐0.14
‐9	‐8.858	‐8.837	‐5.14	32.21	‐2.90	0.06	‐2.96	‐0.09	‐8.78	‐0.07	‐8.881	‐0.10
‐8	‐7.865	‐7.838	‐4.57	32.35	‐2.59	0.05	‐2.64	‐0.08	‐7.83	‐0.03	‐7.888	‐0.06
‐7	‐6.869	‐6.848	‐4.00	32.50	‐2.28	0.04	‐2.31	‐0.07	‐6.87	0.00	‐6.892	‐0.02
‐6	‐5.872	‐5.849	‐3.43	32.65	‐1.96	0.03	‐1.99	‐0.06	‐5.91	0.04	‐5.895	0.01
‐5	‐4.872	‐4.854	‐2.86	32.80	‐1.64	0.02	‐1.66	‐0.05	‐4.94	0.07	‐4.895	0.04
‐4	‐3.876	‐3.845	‐2.29	32.95	‐1.32	0.01	‐1.33	‐0.04	‐3.96	0.09	‐3.899	0.06
‐3	‐2.879	‐2.848	‐1.72	33.10	‐0.99	0.01	‐1.00	‐0.03	‐2.98	0.10	‐2.901	0.08
‐2	‐1.874	‐1.845	‐1.15	33.25	‐0.67	0.00	‐0.67	‐0.02	‐1.99	0.12	‐1.903	0.09
‐1	‐0.869	‐0.853	‐0.57	33.40	‐0.33	0.00	‐0.33	‐0.01	‐1.00	0.13	‐0.887	0.11
0	0.138	0.000	0.00	33.55	0.00	0.00	0.00	0.00	0.00	0.14	0.116	0.12
1	1.147	1.119	0.57	33.70	0.34	0.00	0.34	0.01	1.00	0.14	1.125	0.12
2	2.151	2.117	1.15	33.85	0.68	0.00	0.67	0.02	2.01	0.14	2.129	0.12
3	3.155	3.102	1.72	34.00	1.02	0.01	1.01	0.03	3.02	0.14	3.133	0.11
4	4.157	4.091	2.29	34.15	1.37	0.01	1.35	0.04	4.03	0.12	4.136	0.10
5	5.159	5.091	2.86	34.30	1.72	0.02	1.70	0.05	5.05	0.11	5.137	0.09
6	6.159	6.085	3.43	34.45	2.07	0.03	2.04	0.06	6.07	0.09	6.137	0.07
7	7.158	7.085	4.00	34.60	2.42	0.04	2.39	0.07	7.09	0.07	7.136	0.04
8	8.154	8.074	4.57	34.75	2.78	0.05	2.73	0.08	8.12	0.04	8.132	0.02
9	9.148	9.074	5.14	34.89	3.14	0.06	3.08	0.09	9.14	0.00	9.127	‐0.02
10	10.140	10.063	5.71	35.04	3.51	0.07	3.43	0.10	10.17	‐0.03	10.118	‐0.05
11	11.128	11.063	6.28	35.19	3.87	0.09	3.78	0.11	11.20	‐0.07	11.107	‐0.10
12	12.114	12.052	6.84	35.34	4.24	0.11	4.14	0.12	12.23	‐0.12	12.092	‐0.14
13	13.096	13.052	7.41	35.48	4.62	0.13	4.49	0.13	13.26	‐0.17	13.075	‐0.19
14	14.074	14.041	7.97	35.63	4.99	0.14	4.85	0.14	14.30	‐0.22	14.053	‐0.24
15	15.049	15.041	8.53	35.78	5.37	0.17	5.20	0.15	15.33	‐0.28	15.028	‐0.30
16	16.019	16.030	9.09	35.92	5.75	0.19	5.56	0.16	16.36	‐0.34	15.998	‐0.36
17	16.985	17.030	9.65	36.06	6.14	0.21	5.92	0.17	17.39	‐0.41	16.964	‐0.43
18	17.946	18.019	10.20	36.21	6.53	0.24	6.29	0.19	18.42	‐0.47	17.925	‐0.50
19	18.902	19.019	10.76	36.35	6.92	0.26	6.65	0.20	19.45	‐0.55	18.881	‐0.57
20	19.853	20.008	11.31	36.49	7.31	0.29	7.02	0.21	20.48	‐0.62	19.833	‐0.64

The 50% dose position at 10 MV (column 2) was measured using a nominal light field (column 1). The light field tangential edge position ($X_{\text{tang},p}$ in Fig. 1 and lp in Eq. (1)) (column 3) could be calculated inversely by inputting the 50% of the central axis dose position to replace the $P_{t50}(1p)$ value in Eq. (1). The treatment planning leaf position $\left(X_{\text{mlc},p}\right)$ (column 10) could be calculated by the light field tangential edge $\left(X_{\text{tang},p}\right)$. Offset correction at 10 MV with an SSD of 100 cm was obtained from the 50% dose position minus $X_{\text{mlc},p}$ (column 11). According to the definition of the ADAC treatment planning system, offset is obtained by the value of the 50% dose position minus $X_{\text{mlc}.p}$ of each leaf position at an extended SSD. The offset correction (column 13) at 6 MV with an SSD of 100 cm could be obtained by the same calculation procedures from column 2 to column 10. The results of offset correction at extended SSD at 6 MV and 10 MV are shown in Figs. 6 and 7, respectively. The data for offset correction at different photon energies and extended SSD can be obtained by applying the same procedures described in Table 1.

## V. CONCLUSIONS

It is critical for high‐quality radiation therapy that planned and delivered dose measurement should be at an appropriate level. In this study, we illustrate that the radiation accumulated dose and the planned dose may not always be in agreement for MLC treatment fields at extended SSD unless offset is carefully adjusted.

Calibration could be performed at a certain SSD to fit all SSD offset corrections. For example, at an SSD of 120 cm and the most extreme leaf position settings at +20 cm or ‐12 cm, the offset corrections were 9.9 mm and 3.9 mm at 6 MV and 9.75 mm at 3.7 mm at 10 MV. Patient treatment monitor unit calculations could result in significant underdosage or overdosage if offset correction is not carefully performed in treatment planning. Using the example of film measurements in this study, the monitor unit calculation might recommend an underdosage or overdosage of 7.5% per mm in error offset correction.

If the default rounded leaf position table provided by the treatment planning system is utilized to set the rounded leaf‐end position offset correction, the dose error is between 6% and 8%. With careful measurement and an accurate offset correction, it is possible to achieve dose calculation within 0.5% error for the adjusted MLC leaf edge location in the treatment planning system.

## ACKNOWLEDGMENTS

The authors thank the anonymous reviewers for their helpful comments on the original manuscript. This study was supported by grants from E‐Da Hospital (EDAHT101007) and NSC 101‐2221‐E‐151‐007‐MY3.

## APPENDIX A: TRANSMISSION PENUMBRA

Schematic drawing of a mathematical model for deriving the 50% dose position ($P_{t50}$) and the leaf edge position of the tangential field $\left(X_{\text{tang},p}\right)$ at a certain SSD. The quantified field edge precise position $\left(X_{\text{tang},p}\right)$ is transformed purposely from a nominal light field edge which was used for the measurement of the 50% dose position. Leaf position is denoted as “lp” in this figure and is the same as leaf edge position of the tangential field $\left(X_{\text{tang},p}\right)$ in Fig. 2.

The analytical formula of transmission penumbra depending on leaf position is presented in this Appendix. In this figure, a schematic view of a leaf from the right bank placed at the right edge of a field is shown. If a leaf position (*lp*) in the field space is known, a ray line through which irradiation will drop to 50% from the initial irradiation can be defined.

The figure shows all variables relevant for finding position of point $Pt_{50}(lp)$,

$$Pt_{50}(lp)=F\cdot \tan(\gamma_{50(lp)})$$

where $\gamma_{50(lp)}$ is an angle between 50% ray line and the central axis,

$$\gamma_{50(lp)}=\alpha_{\left(lp\right)}-\delta_{50(lp)},$$

where $\alpha_{\left(lp\right)}$ is an angle between ray line connecting center of a tip (point O) curvature and the central axis, and $\delta_{50(lp)}$ is an angle between 50% ray line and the center tip ray line,

$$\alpha_{\left(lp\right)}=\arctan\;(MO/c),\delta_{50(lp)}=\arcsin\;(h_{50(lp)}/a_{\left(lp\right)})$$

where $\bar{MO}$ is the distance between the center of a tip (point O) and the central axis, $a_{\left(lp\right)}$ is the distance between the source and the center of tip (point O), *c* is the distance source center of a leaf and $h_{50(lp)}$ is the distance between 50% ray line and the center of the tip,

$$\begin{array}{rl} & h_{50(lp)}={\left[R^{2}-{\left(d_{50}/2\right)}^{2}\right]}^{1/2}\\ & d_{50}=-\ln(0.5)/\mu \end{array}$$

where *R* is a radius of the tip, $d_{50}$ is a path length through the tip and μ is a coefficient of attenuation.

Further, from equation above, $a_{\left(lp\right)}$ and *MO* are $a(lp)={\left(MO^{2}+c^{2}\right)}^{1/2}$,

$$MO=c\cdot (lp/F)+x_{\left(lp\right)}+l_{\left(lp\right)}.$$

Above figure shows variables $x_{\left(lp\right)}$, $y_{\left(lp\right)}$ and $l_{\left(lp\right)}$,

$$\begin{array}{rl} & l_{\left(lp\right)}=y_{\left(lp\right)}\cdot (lp/F)(distance\;between\;m\;and\;d\;in\;above\;figure)\\ & y_{\left(lp\right)}=R\cdot \sin(\theta )(distance\;between\;C\;and\;d\;in\;above\;figure)\\ & x_{\left(lp\right)}=R\cdot \cos(\theta )(distance\;between\;d\;and\;O\;in\;above\;figure).\\ & c\cdot (lp/F)=Mm(MO=Mm+md+dO) \end{array}$$

Finally, Θ is an angle between the tangent of a leaf tip and the central axis

Θ=arctan⁡(lp/F)

Now, by substitution of all variables together to form the position of the point $Pt_{50(lp)}$ is given in Eq. (1).

## Supporting information

Supplementary Material Files
