# Supplementary figures and images for: A light field‐based method to adjust rounded leaf end MLC position for split shape dose calculation correction in a radiation therapy treatment planning system
Source: J Appl Clin Med Phys. 2012 Nov 8;13(6):3–18. doi: 10.1120/jacmp.v13i6.3937 (PMC5718526; doi:10.1120/jacmp.v13i6.3937)

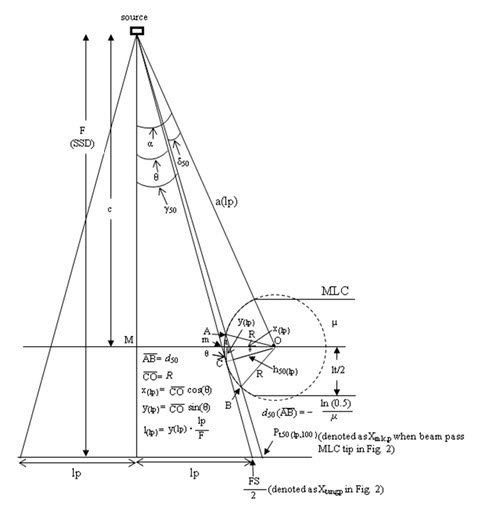

Supplement: Supplementary file 1 — Supplementary Material Files [file ACM2-13-003-s001.jpg]
